# Acute rehabilitation following traumatic anterior shoulder dislocation (ARTISAN): pragmatic, multicentre, randomised controlled trial

**DOI:** 10.1136/bmj-2023-076925

**Published:** 2024-01-17

**Authors:** Rebecca S Kearney, David R Ellard, Helen Parsons, Aminul Haque, James Mason, Henry Nwankwo, Helen Bradley, Stephen Drew, Chetan Modi, Howard Bush, David Torgerson, Martin Underwood, The ARTISAN collaborators, Kerri McGowan, Joanna Brough, Howard Bush, Chetan Modi, Oliver Donaldson, Michael Jones, Alison Lewis, Kate Beesley, Cheryl McDermott, Caroline Bennett, Joyce Guy, Jospeh Askew, Charlie Talbot, Dave Copas, Su Leeming, Angela Sheldon, Venkata Vakamallu, Erin Demoulin, Tom Bradshaw, Deborah Wilson, Emma Tindall, Andrea Meddes, Rajesh Nanda, Jane Evans, Steven Barnfield, Kirsty Mapstone, Rhian Witham, Ian Packham, Paula Brock, Josephine Morley, Fionnaula Palmer, Ruth Halliday, Katherine Coates, Laura Lunny, Vicki Stoneman, Nicola Ward, James Kennedy, Celia Whitehouse, Carol Payne, Emmet Griffiths, Andrew Lee, Leila Dehghani, Aileen Nacorda, Natasha Woodbine, Niel Kang, Kelly Marie Grant, Laura Fabb, Rebecca Lewis, Marcus Bateman, Evelina Siwerska, Louise Amandini, Caroline Clarkson, Kaite Walstow, Emma Whitby, Rebecca Smith, David Clark, Mona Mohamed, Kathleen Holding, Leicester Royal Infirmary, Kathryn Robinson, Tracey Lee, Helen Fort, Alison Armstrong, Amber Bery, Paul Lewis, Dileep Kumar, Helen Tunnicliffe, Royal Victoria Infirmary, Karen Smith, Jenny Baron, Lisa Robinson, John Williams, Heather Hunter, Anne Marie Ginnever, John Paul Gowland, Sophie Collins, Nicola Rose, Kate Graham, Helen Watson, Stephen Aldridge, Steven Galloway, Stuart Watson, Hannah Riley, Kathryn Hanson, Simon Fogerty, Laura Hedley, Tash Maher, Siobhan Currie, Kayleigh Leatham, Andrew White, James Wheble, Susan O'Sulivan, Stephanie Diaz, Paula Goodyear, Janki Bhayani, Deborah Butcher, Nicola Parker, Richard Cooper, Lisa Armstrong, Emma Dooks, Stacey Lalande, James Tyler, Sally Mitchell, Chantel Mcparland, Natalie Rose Craven, Lauren Harker, Jessica Holdsworth, Martin Thomas, Zanib Taj, Daniel Kantas, Justin Murr, Rob Hunter, Rohit Gupta, Sebastian Prentice, Leon Palmerwilson, Caroline Coulthard, Nicky Holland, Isaac John, Kathryn Lewis, Warren Sheehan, Colin Forde, Tessa Sedwin, Chandramohan Panneerselvam, Caroline Partner, Pablo Llorian, Georgina Taylor, Maria Mestre, Andrew Titchener, Sangeetha Prasath, Ella Riedel, Alex Maley, Emma Stoddard, Charalambos P Charalambous, Suzanne Lane, Clare Blundell, Conor Wilkinson, Gemma Brown, Sarah Melling, Marin Sirbu, Sam Remnant, Shamina Hankinson, Denise Bennett, Emma Ward, Sean Dixon, Sharon Botfield, Cathal Murphy, Suzanne Dean, Eve Fletcher, Deanne De Beer, Annie Rae, Andrew McCarley, Hayley Potter, Damian Warner, Tamsin Ranson, Steven Cromey, Nicole Toomey, Jennifer Armistead, Kelly Brooks, Leanne Dymott, Chloe Brown, Georgie Parsons, Salma Chaudhury, Terence Campbell, Emma Craig, Joanna Pinto, Rashmi Easow, Amar Malhas, Catherin Anderson, Greg Quinn, John Whitwell, Nadine Siddle, Mary Wragg, Hywell Williams, Paula Strider, Rebecca Tait, Clive Nicholson, Liz Johnson, Pearl Clark Brown, Scott Maccines, David Bailey, Gemma Kirkman, Lisa Warren, Julie Bury, Lisa Mellish, John Rice, Rhys Owen, Christopher Greenfield, Ajay Sharma, Joanna Wilson, Caroline Hamilton, Scott Barker, Carol Carnegie, Ann Quirk, Claire Stewart, Bally Purewal, Deborah Beeby, Stephanie Bell, Carol Buckman, Stephanie Garnham, Chris Roberts, Marie Welch, Fiona Carleton, James Doonan, Katharine Duffy, Valerie Forgie, Paul Jenkins, Jennifer Love, Linda Mercer, Gary Semple, Angharad Williams, Thomas Lockwood, Yemi Pearse, Sarah Latham, Gemma Shearer, Susan Nickle, Teresa Cepelkova, Andrew Wright, Kirsty Harwood, Michael Flatman, Miles Benjamin, Alexandros Haris, Oliver Brown, Prodrollos Tsinaslendis, Mathew Facey, Bethany Hedges, Abrar Gani, Selma Al-Ahmad, Nicholas Judkins, Judith Burnham, Andrew Caukroger, Tomisin Omogbehin, Conor McDermott, Cleo Dobson, Charlotte Brookes, Sara Dardak, Roshan Rupra, Rhody Asirvatham, Maria Dadabhoy, Jennifer Rees, Sally Fisher, Simon Nicole, Lynn Smyth, Kathryn Allison, Ellie Higham, Jayne Craig, Jack Burke, Sarah Hart, Allie Menzies, Deborah Power, Holly Davies, Alisha Walters, Kerry Simpson, Karen Burns, Laura Dodd, Gautam Talawadekar, Syed Mannan, Scott Smith, Helen Wilson, Melanie Clapham, Jane Gregory, Steven Wright, Una Poultney, Polly Rice, Nicci Kelsall, Katherine Davidson, Fran Armstrong, Diane Armstrong, David Mackay, Hannah Craig, Natalie Keenan, Matt Hurst, Maureen Holland, Jessica Clitheroe, Jochen Fischer, Victoria Austin, Jack Mullins, Charles Corbin, Sarah Shephardson, Alison Wilkinson, Rosaline Keane, Carol Lockwood, Helen Morris, Lynn Osbome, Alun Yewlett, Jo Fletcher, Eleanor Gates, Grace Homer, Kimberley Netherton, Munya Tsuro, Alun Yewlett, Simon Booker, Valerie Jones, Grahame Clark, Bonito Tsong, Abiola Alli, Rachel Sellars, Julie Walker, Elizabeth Hurditch, Pene Fati, Karne Robinson, Harjinder Kaur, Sandra Guzaviciute, Louise Mew, Lee How, Annie Rose, Louise Kelly, Louise Heylen, James Underwood, Aabid Sanaullah, Sarah Overall, Yan Cunningham, Julie Doughty, Yusuf Michla, Debbie Watson, Royal Devon, Peter Hamer, Adel Tavakkolizadeh, Nicky Wilson, Victoria Rowe, Christopher Ratcliffe, Scarlett McCalla, Ines Reichert, Kerim Gokturk, Hazel Giles, Victoria Withers, Christy Walkin, Harshinder Virdee, Raye Marinas, Laarni Bonganay, Sheena Mendoza, Christopher Beggs, Gary Chan, Jordan Bethel, Silvana Skotiniyadis, Ross Moorely, Barnaby Sheridan, Simon Ingram, Kimberley Gillman, Alison Whitcher, Claire Gutherie, Lynne Connell, Carole Cummings, Pascal De Feyter, Nicola De Jong, Gary Dobson, Amy Moreton, Layla Morissey, Nasir Shah, Suzi Thompson, Rachel Dean, Saif Hadi, Karen Marsden, Helen Thompson, Philip Ahrens, Andrea Francis, Fracnesca Gowing, Rebecca Portwood, Nikunji Depala, Stamatios Tsamados, Adam Yasen, Lara Beaman, Sarah Flynn, Sangna Unadkat, Aiden Gallagher, Lisa Mellish, John Rice, Rhys Owen, Christopher Greenfield, Ajay Sharma, Joanna Wilson, Caroline Hamilton

**Affiliations:** 1 Bristol Trials Centre, University of Bristol, Bristol, UK; 2 Warwick Clinical Trials Unit, University of Warwick, Warwick, UK; 3 University Hospitals Coventry and Warwickshire NHS Trust, Coventry, UK; 4 York Clinical Trials Unit, University of York, York, UK

## Abstract

**Objective:**

To assess the effects of an additional programme of physiotherapy in adults with a first-time traumatic shoulder dislocation compared with single session of advice, supporting materials, and option to self-refer to physiotherapy.

**Design:**

Pragmatic, multicentre, randomised controlled trial (ARTISAN).

**Setting and participants:**

Trauma research teams at 41 UK NHS Trust sites screened adults with a first time traumatic anterior shoulder dislocation confirmed radiologically, being managed non-operatively. People were excluded if they presented with both shoulders dislocated, had a neurovascular complication, or were considered for surgical management.

**Interventions:**

One session of advice, supporting materials, and option to self-refer to physiotherapy (n=240) was assessed against the same advice and supporting materials and an additional programme of physiotherapy (n=242). Analyses were on an intention-to-treat basis with secondary per protocol analyses.

**Main outcome measures:**

The primary outcome was the Oxford shoulder instability score (a single composite measure of shoulder function), measured six months after treatment allocation. Secondary outcomes included the QuickDASH, EQ-5D-5L, and complications.

**Results:**

482 participants were recruited from 40 sites in the UK. 354 (73%) participants completed the primary outcome score (n=180 allocated to advice only, n=174 allocated to advice and physiotherapy). Participants were mostly male (66%), with a mean age of 45 years. No significant difference was noted between advice compared with advice and a programme of physiotherapy at six months for the primary intention-to-treat adjusted analysis (between group difference favouring physiotherapy 1.5 (95% confidence interval −0.3 to 3.5)) or at earlier three month and six week timepoints. Complication profiles were similar across the two groups (P>0.05).

**Conclusions:**

An additional programme of current physiotherapy is not superior to advice, supporting materials, and the option to self-refer to physiotherapy.

**Trial registration:**

Current Controlled Trials ISRCTN63184243.

## Introduction

The shoulder is the most frequently dislocated joint.^1^ Dislocation happens when excessive forces during a traumatic event displace the humeral head out of the shoulder socket resulting in the joint surfaces completely losing contact.^2^ ^3^ ^4^ In 95% of cases, the shoulder dislocates anteriorly (forwards). After a shoulder dislocation has occurred, emergency treatment is required to reduce the dislocation and to support the arm in a sling.^2^

A 2019 population based cohort of 16 763 people reported the incidence to be 40.4 per 100 000 person years in men and 15.5 in women.^1^ The highest incidence was in men aged 16-20 years (805 per 100 000 person years) with a second peak in women aged 61-70 years (28.1 per 100 000 person years).^1^ The first peak is attributed to sporting injuries, the second peak is associated with injuries incurred during a fall in an ageing population.^5^

Non-operative rehabilitation care is the most common first line treatment, typically involving the arm being supported in a sling for up to two weeks, for comfort and to allow healing.^3^ This treatment is followed by rehabilitation for up to six months.^2^ Rehabilitation helps support people to restore a functional, painless shoulder and is achieved through restoration of movement and retraining muscles.^3^

During this period of rehabilitation, people experience pain and disability due to decreased movement of the arm. As such, simple tasks, such as getting dressed, can become difficult.^2^ ^3^ ^4^ Some people also experience a constant sensation that the shoulder is going to dislocate again, which stops them from returning to some activities. Within 12 months, one in five people will re-dislocate their shoulder, leading to repeated visits to the emergency department and ongoing pain.^6^

Despite the important role of rehabilitation for this common condition, evidence comparing different rehabilitation methods after the initial period in a sling is scarce.^2^ ^4^ ^7^ Guideline recommendations internationally range from advice only to advice and an additional programme of supervised physiotherapy.^2^ ^4^

The choice of rehabilitation offered following a shoulder dislocation has large resource implications for the participant and health care provider. Younger people may need to take time from work or arrange care for dependents and older people may find travel challenging particularly if unable to drive following the dislocation. Consequently, if a single advice session were all that is required, the burden on patients and the healthcare resources required would lessen.

Considering the large personal and societal cost associated with this injury, the primary objective of the ARTISAN trial was to compare the clinical effectiveness of two rehabilitation interventions in adults with a first time traumatic shoulder dislocation.

## Methods

### Study design

This pragmatic, superiority, randomised multicentre controlled trial was conducted at 41 UK hospital trusts in the National Health Service (NHS). The national research ethics committee approved this study on 26 July 2018 (18/WA/0236), with each trial site granting individual NHS Trust approval before recruitment at each site. The protocol and statistical analysis plan were approved by the independent trial steering committee and data and safety monitoring committee. These independent committees were convened to oversee the study throughout the trial duration. The ARTISAN protocol was accepted for publication on October 13 2020 and first published on November 19 2020.^8^ The statistical analysis plan is available in Supplement 1. The ARTISAN study was prospectively registered on 7 September 2018 (ISRCTN63184243).

### Participants

Trauma research teams at 41 UK NHS Trust sites screened adults who had a traumatic anterior shoulder dislocation for the first time that had been confirmed radiologically and who were being managed non-operatively from orthopaedic clinics. Participants were excluded if they had neurovascular complications, bilateral dislocations at the time of injury, had been randomly assigned previously in this study, were unable to adhere to trial procedures, or were unable attend physiotherapy within six weeks of injury. After assessment, potential participants were provided with verbal and written information before they provided written informed consent.

### Randomisation

All baseline data were collected before randomisation. At the end of the initial advice session (provided to all participants), participants were randomly assigned to no further treatment (advice only) or the offer of further physiotherapy. Allocation concealment was maintained by an independent randomisation team at Warwick Clinical Trials Unit who were responsible for generation of the sequence and had no role in participant recruitment. The treatment groups were randomised strictly sequentially on a 1:1 basis. Participants were allocated by a secure web based system using a minimisation algorithm with a random element and stratification by centre, participant age (≤39 years and ≥40 years), and whether the dominant arm was injured. These factors were included to account for any centre affects, the association of age with higher risk of re-dislocation, and any association of arm dominance on self-reported measures of function.

Following randomisation, masking participants or treating clinicians to treatment allocation was not possible. However, the treating clinician and the participant were masked to treatment allocation during the initial advice session. The central research team members were masked until after data analysis was complete, except for the trial statisticians, who had access to treatment assignment for the purposes of data monitoring and safety, and data entry personnel, who entered data from questionnaires, including some details of treatments received.

### Interventions

All participating sites received an initial training session from an ARTISAN trial research physiotherapist. Following this, a lead physiotherapist at each site was identified to complete subsequent training of additional physiotherapists. This training was supported with web based materials and a trial intervention manual. Intervention fidelity was monitored by direct observations, audio recordings of the advice session (twice annually per site), and physiotherapy checklist self-reports of the advice session (all participants).

All participants had an initial period in which the injured arm was supported in a sling and then received an appointment for a physiotherapy advice session within six weeks of their injury. At the first appointment, all participants received the same initial shoulder examination followed by advice to aid self-management, lasting up to one hour and administered by an ARTISAN trained physiotherapist. This session included core components on education, progressive exercises, and exercise planning to enhance self-management behaviours. These core components were available after the advice session via a password protected website or via paper booklet, depending on participant preference. Details of the intervention development were first published in December 2021.^9^

Following completion of the advice appointment, the participant was randomly assigned to only this advice session or to this advice session plus the offer of additional physiotherapy. Participants randomly assigned to advice only were provided with a contact point to self-refer back to the clinical team if recovery did not occur. Participants who self-referred back to the clinical team were considered to be per protocol.

Participants randomly assigned to the additional intervention group offered additional physiotherapy sessions. Each additional session lasted for up to 30 min, over a maximum duration of four months from the date of randomisation. The course of physiotherapy involved teaching and supervising the core set of progressive exercises offered to the control group in addition to being able to tailor treatment according to usual practice.

### Outcome measures

The primary outcome was the Oxford shoulder instability score at six months. This score is a self-completed outcome measure containing 12 questions (0-4 points each), with possible scores from 0 (worst function) to 48 (best function).^10^ ^11^ Questions relate to activities of daily living that are particularly relevant to patients who have shoulder instability and they have been designed to assess outcome of treatment.

Secondary outcomes included QuickDASH, EQ-5D-5L, and any complications.^12^ ^13^ QuickDASH is a shortened version of the disabilities of the arm, shoulder and hand (DASH) questionnaire. The tool uses 11 items to measure physical function and symptoms in people with any musculoskeletal disorder of the upper limb. We also measured the Oxford shoulder instability score at six weeks, three months, and 12 months.

Secondary outcome measurements were collected at six weeks, three months, six months, and 12 months by postal questionnaire. Six months was the primary outcome, in keeping with national guidelines on duration for rehabilitation of this injury. Telephone follow-up was used to contact people who did not respond or fully complete the returned postal questionnaire.

### Statistical analysis

The target between-group difference that was considered worthwhile for the primary Oxford shoulder instability score outcome score was 4 points.^14^ ^15^ The standard deviation of the Oxford shoulder instability score six months after injury was previously reported as 10 points.^14^ ^15^ However, the literature had predominantly included a younger population than the one that we planned to recruit, therefore, with our wider range of ages, a larger standard deviation was expected, so a standard deviation of 12 was used. As such, 478 participants were required to show a 4 point target difference (ie, a small, standardised mean difference of 0.3) at the 5% significance level, with 90% power, allowing a margin of 20% loss during follow-up.^11^ ^15^

Analyses were on an intention-to-treat basis with secondary per protocol analyses. The main analysis investigated differences in the primary outcome measure between the two treatment groups, six months after randomisation. Unadjusted and adjusted mixed effects linear regression models were used to estimate the between group difference. The adjusted analyses included the stratification variables (centre, participant age, group, and if the dominant arm was injured) and baseline scores, except for complications, which were only compared using Fisher’s exact test (ie, unadjusted analysis only). Multiple imputation was used to assess the effects of missing data. Analyses were conducted in R (version 4.0.3) and a detailed statistical analysis plan was written before any formal analyses and was approved on 25 October 2021.

Since individual clinicians would treat only a small number of participants enrolled in the trial, we did not expect clinician specific effects to be important in this study but recognised the theoretical possibility of therapist effects. To address this, a single interim analysis was preplanned to re-estimate the sample size. This analysis occurred after 200 participants had completed the three month follow up questionnaire, while recruitment was still open.

The pooled standard deviation of the primary outcome and presence of therapist effects was estimated by calculating the therapist intraclass correlation coefficient using a mixed effect model containing site of randomisation, baseline Oxford shoulder instability score, age group, and if the participant’s dominant arm was injured as fixed effects. The treating physiotherapist was then added as a random effect. The 95% confidence interval of the intraclass correlation coefficient was then calculated using bootstrap methods. Models did not include treatment effects.

We used two prespecified exploratory subgroup analyses: hand dominance (injured shoulder dominant arm *v* injured shoulder non-dominant arm) and age (younger participants *v* older participants). The subgroup analyses followed the methods described for the primary analysis, with additional interaction terms incorporated into the mixed effects regression model to assess the level of support for these hypotheses.

### Patient and public involvement

Before the study, clinical co-applicants consulted with patients during appointments to ascertain if the research gaps highlighted in the literature were of high importance to them. Once the importance of the topic was established, a patient group was convened to discuss experiences and expectations of rehabilitation services and the plans for the trial. These perspectives were key in the development of the protocol to ensure trial processes, materials, and interventions were feasible and acceptable.

Subsequently, a patient representative was included as a lay co-applicant who in addition to contributing during our development work, was a member of the trial management group. They contributed to trial processes, such as reviewing patient facing materials. A further patient representative agreed to part of the independent trial steering committee for the duration of the trial and advised on the final report and dissemination plans.

## Results

### Participants and adherence

We screened from 41 UK NHS Trusts, and recruited from 40, between 14 November 2018 and 14 March 2022. Trusts screened 1551 adults with a traumatic shoulder dislocation. Of these, 922 patients were eligible after initial screening in the orthopaedic clinic. All participants were then referred to their initial physiotherapy advice consultation where a further eligibility screen was undertaken, at which stage, 77 people were deemed ineligible. Overall, 482 were randomly assigned to either advice only (n=240) or to advice and a programme of physiotherapy (n=242).

**Fig 1:**
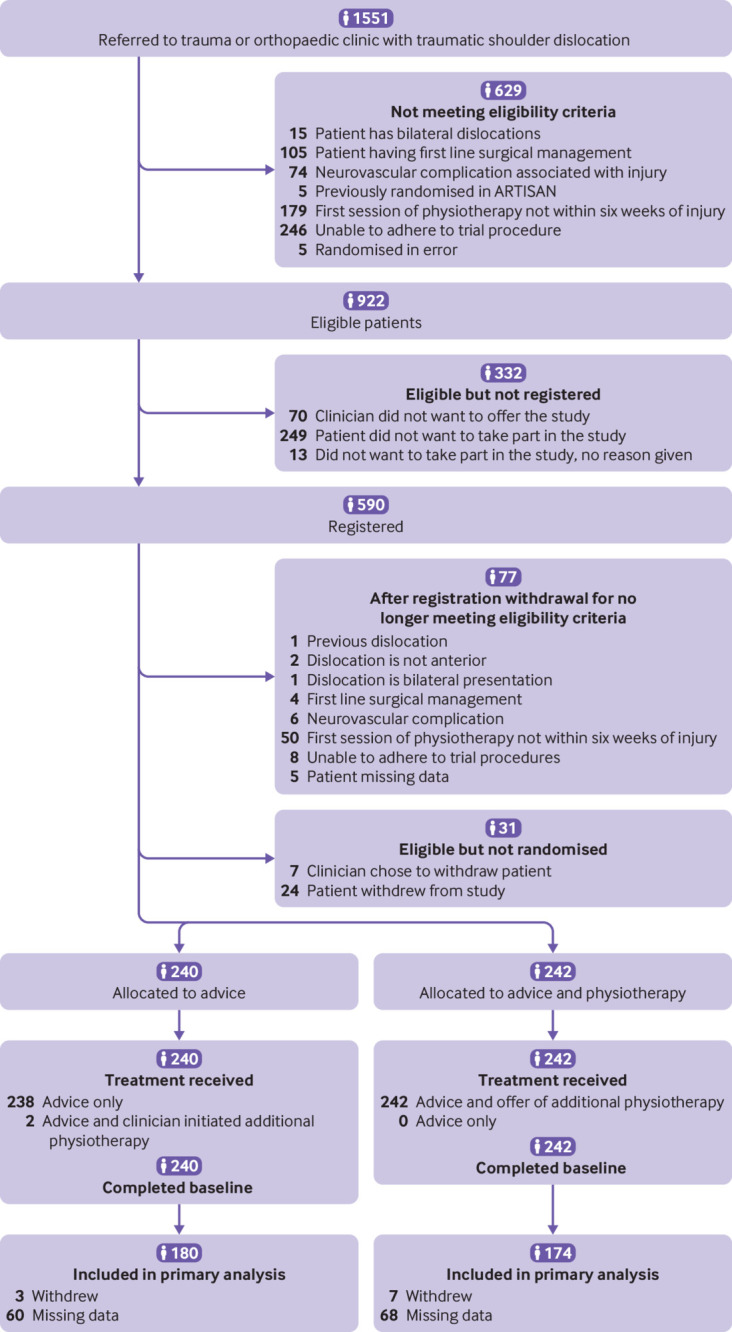
Recruitment, randomisation, and follow-up in ARTISAN

Ten participants withdrew before the primary outcome point of six months; 354 participants completed the primary outcome Oxford shoulder instability score (73%) and were included in the final analysis (fig 1). Participants were mostly male (66%, n=317), with a mean age of 45 years. The groups were well balanced across baseline characteristics (table 1).

**Table 1:** Descriptive characteristics of population by allocated treatment group at baseline*. Number of participants (percentage), unless otherwise specified

Characteristics	Advice (n=240)	Advice and physiotherapy (n=242)	Overall (n=482)
Sex:			
Male	158 (66)	159 (66)	317 (66)
Female	82 (34)	83 (34)	165 (34)
Race and ethnicity/ancestry†:			
Asian	18 (8)	19 (8)	37 (8)
Black/African/Caribbean	12 (5)	5 (2)	17 (4)
Mixed	2 (1)	7 (3)	9 (2)
Other	3 (1)	5 (2)	8 (2)
White	205 (85)	206 (85)	411 (85)
Age, mean (SD):	45 (20)	44.7 (19)	44.9 (19.6)
≤39 years (%)	109 (45)	112 (46)	221 (46)
≥40 years (%)	131 (55)	130 (53)	261 (54)
Injured arm:			
Dominant	134 (56)	136 (56)	270 (56)
Non-dominant	106 (44)	106 (44)	212 (44)
Mechanism of injury:			
Sports	94 (39)	78 (32)	172 (36)
Not sports	146 (61)	164 (68)	310 (64)
Concurrent injuries:			
No	190 (79)	196 (80)	386 (80)
Yes	50 (21)	46 (19)	96 (20)
Regularly smokes:			
No	209 (87)	198 (82)	407 (84)
Yes	31 (13)	44 (18)	75 (16)
Alcohol units (per week):			
0-7 units	154 (64)	157 (65)	311 (65)
8-14 units	57 (23)	53 (22)	110 (23)
15-21 units	14 (6)	21 (9)	35 (7)
>21 units	14 (6)	11 (5)	25 (5)
Taking other medication:			
Steroids	2 (1)	5 (2)	7 (2)
Any other pain medications	19 (8)	22 (9)	41 (9)
Diagnosis before injury:			
Diabetes	9 (4)	9 (4)	18 (4)
Inflammatory arthritis	8 (3)	6 (3)	14 (3)
Employment status:			
Full time employed	119 (50)	113 (47)	232 (48)
Part time employed	26 (11)	20 (8)	46 (10)
Self-employed	14 (6)	31 (13)	45 (9)
Retired, Student or other	50 (21)	48 (20)	98 (20)
Unemployed	11 (5)	8 (3)	19 (4)
Unpaid work	0 (0)	1 (<1)	1 (<1)
Full time student	18 (8)	21 (9)	39 (8)
Full time carer	2 (1)	0 (0)	2 (<1)

SD=standard deviation. *Less than 3% missing in any category. †Ethnic group was self-reported using the listed options, with participants only able to select one option; no participants reported Chinese or Bangladeshi ethnic group. “Other” was included as a category from which participants could choose.

Ninety six physiotherapists delivered the interventions across the 40 sites (supplement 2, table 1). Adherence was high across the groups. In the advice only group, adherence was 98% (n=236) with 81% (n=194) of 240 participants receiving advice only and 18% (n=42) of participants self-referring to receive a programme of physiotherapy. Only 1% (n=2) of participants in the advice only group crossed over to receive a programme of physiotherapy due to the clinician’s recommendation. Data were unobtainable for two participants (supplement 2, figure 1).

In the group randomised to advice and further physiotherapy, adherence was 100% (n=242), with all participants offered additional physiotherapy. Within this group, 69% (n=167) of 242 participants had a complete programme of physiotherapy, defined as completing all sessions scheduled over the four month period, 10% (n=24) of participants did not attend any additional appointments, and 12% (n=30) of participants did not attend after one appointment. After the four month treatment period, 7% (n=18) of participants were receiving ongoing management (supplement 2, figure 2).

The intraclass correlation coefficient at the interim analysis point (at three months: 138 participants and 67 physiotherapists) was estimated to be 0.0201 (95% confidence interval 0 to 0.601). The oversight committees agreed that no adjustment to our target sample size was needed. Repeating this analysis using the six month data at the end of the study gave an intraclass correlation coefficient of 0.026 (95% confidence interval 0 to 0.106).

### Outcomes

No significant difference in Oxford shoulder instability scores were reported between the two groups at the six month primary outcome (mean 25 weeks (standard deviation 4)), for the primary intention-to-treat adjusted analysis (mean difference favours physiotherapy 1.5 (95% confidence interval −0.3 to 3.5)) or at earlier three month and six week time points (table 2). At all timepoints, the direction of change favoured a programme of physiotherapy; however, the 95% confidence interval at each time point excluded the target (worthwhile) 4 point difference. For secondary outcomes, no significant differences were reported in the QuickDASH or consistent differences shown from the EQ-5D-5L (table 3).

**Table 2:** Oxford shoulder instability score (0 to 48, higher scores indicate better function) in the intention-to-treat population, positive value in favour of advice and physiotherapy

Time	Advice (n=240)		Advice and physiotherapy (n=242)		Between group difference (95% CI)	
	n	Mean (SD)	n	Mean (SD)	Adjusted*	P value
6 weeks	166	23.3 (10.4)	173	24.4 (9.9)	0.7 (−1.0 to 2.4)	0.44
3 months	170	30.0 (11.4)	182	32.2 (10.4)	1.6 (−0.5 to 3.6)	0.13
6 months	180	36.2 (10.7)	174	38.4 (9.2)	1.5 (−0.3 to 3.5)	0.11
12 months	129	39.9 (9.2)	135	41.6 (7.8)	1.1 (−0.9 to 3.1)	0.29

CI=confidence interval; SD=standard deviation. *The model has been adjusted for fixed effects of arm dominance, age group, and baseline scores. Site of randomisation was fitted as a random effect

**Table 3:** Secondary outcomes, QuickDash (0 to 100, higher score means greater disability) and EQ-5D-5L (−0.594 to 1, higher scores mean better health) in the intention-to-treat population, with a positive value in favour of advice and physiotherapy

Secondary outcomes	Advice (n=240)		Advice and physiotherapy (n=242)		Between group difference (95% CI)	
	n	Mean (SD)	n	Mean (SD)	Adjusted*	P value
QuickDash:						
6 weeks	154	32.8 (23.2)	168	27.6 (21.4)	−1.9 (−5.5 to 1.7)	0.31
3 months	162	22.8 (21.7)	177	19.3 (19.9)	−1.6 (−5.3 to 2.1)	0.39
6 months	169	14.4 (17.5)	169	12.7 (16.9)	0.8 (−4.0 to 2.5)	0.65
12 months	126	11.0 (16.0)	133	9.2 (15.2)	−0.7 (−4.1 to 2.6)	0.67
EQ-5D-5L:						
6 weeks	166	0.692 (0.201)	173	0.711 (0.189)	0.004 (−0.030 to 0.038)	0.81
3 months	170	0.741 (0.215)	182	0.787 (0.179)	0.030 (−0.005 to 0.064)	0.10
6 months	179	0.797 (0.217)	173	0.815 (0.183)	0.010 (−0.026 to 0.047)	0.59
12 months	129	0.848 (0.169)	135	0.87 (0.16)	0.009 (−0.26 to 0.047)	0.60

CI=confidence interval; SD=standard deviation. *The model has been adjusted for fixed effects of arm dominance, age group and baseline scores. Site of randomisation was fitted as a random effect.

Secondary unadjusted and per protocol analyses, and a sensitive analysis accounting for missingness (multiple imputation), were not different (table 3); although, people lost to follow up at six months were more likely to be younger than 40 years and male. Loss to follow up was similar between allocation groups. Our predefined subgroup analyses for age group (≤39 years and ≥40 years) and arm dominance showed little effect on outcome (supplement 2, figure 3, table 4, and table 5).

### Adverse events

Complication profiles were similar across the two groups and no significant differences were noted. Prespecified expected complications in the advice group after randomisation, included reports of 22 rotator cuff tears, eight compression fractures of the shoulder, seven shoulder re-dislocations, three frozen shoulders, and one report of nerve damage (table 4). In the additional physiotherapy group, 21 rotator cuff tears, seven frozen shoulders, four compression fractures of the shoulder, and three shoulder re-dislocations were reported.

**Table 4:** Analysis of secondary outcome complications from baseline to 12 months (intention-to-treat population)

Complication	Advice (n=240)	Advice and physiotherapy (n=242)	P value*
Torn rotator cuff	22 (9)	21 (9)	0.87
Shoulder re-dislocation	7 (3)	3 (1)	0.22
Frozen shoulder	3 (1)	7 (3)	0.34
Compression fracture	8 (3)	4 (2)	0.26
Ongoing nerve damage	1 (<1)	0 (0)	1.00

Numbers shown are complications reported at least once per participant.

* Fisher’s exact test.

## Discussion

### Principal findings

ARTISAN is the largest randomised controlled trial investigating two different rehabilitation approaches in adults with a first time traumatic shoulder dislocation. We did not report a difference in the mean primary outcome (Oxford shoulder instability score) at the primary outcome time point of six months. Furthermore, because the 95% confidence intervals of the estimate of effectiveness excludes the prespecified worthwhile difference of 4 points, the additional physiotherapy programme was not a worthwhile benefit.

No clinically relevant differences in Oxford shoulder instability score were reported at secondary timepoints or in the secondary outcome measures. We found no significant differences in complications across both interventions.

Until ARTISAN, no strong evidence was available to guide rehabilitation management following an initial two weeks support in a sling. We now know an additional programme of individually tailored physiotherapy is not superior to advice, supporting materials, and an option to self-refer to physiotherapy.

### Comparison with other studies

A 2014 Cochrane review on methods of non-operative management showed no randomised controlled trials comparing different rehabilitation methods after the initial two weeks of supporting the arm in a sling. The review also had no evidence of any ongoing studies.^3^ An updated review in 2019 had the same conclusion, but identified one ongoing study, in addition to this study, that has since been completed.^3^ ^7^ No further ongoing studies were identified in an updated search of trial registries .

The identified randomised controlled trial randomly assigned 56 participants, across three orthopaedic shoulder units in Denmark to a home based exercise intervention or to a physiotherapist supervised 12 week intervention.^16^ This trial included participants younger than 40 years only, whereas ARTISAN included all ages. The authors reported that adherence was low in the supervised physiotherapy group, with only 43% of participants compliant (n=12), but a significant improvement was noted in the primary outcome (western Ontario shoulder instability index) from supervised exercise when compared with home exercises. The point estimate was less than their predefined, clinically relevant, between group difference of 250, although a clinically relevant difference was not excluded (between group mean difference −228.1 (95% confidence interval −430.5 to −25.6); P=0.028).

In ARTISAN, although the point estimate favoured physiotherapy, overall, the result was not significant, and the limits of the 95% confidence interval excluded our target difference thereby showing that that the additional physiotherapy did not have a worthwhile clinical effect.

The overall occurrence of shoulder re-dislocation in this study was 10 (2%) of 482 participants. This number is low in comparison to previous observational cohort data.^6^ However, the follow up timepoint for ARTISAN was six months, whereas the risk of re-dislocation continues beyond this timepoint. The ARTISAN study also included all ages, which may have also been a factor.

The advice only intervention was delivered by physiotherapists and crucially did not prohibit patients from self-referring back to the service if recovery did not meet patient expectations. With this mechanism in place, 18% (42/240) self-referred back to the service. Empowering people to make their own treatment decisions was acceptable to clinicians (98% adherence) and allowed flexibility for people recovering from a first-time shoulder dislocation to decide when additional supervised treatment was required. We acknowledge that additional supervised physiotherapy will be appropriate in some circumstances, however, as a default referral pathway, this treatment option is not superior to a single session of advice, supporting materials, and option to self-refer to physiotherapy intervention.

### Limitations

The main limitation was the 27% loss to follow up, however, the observed standard deviation was much smaller than anticipated. This change in parameters reduced the number of participants required to observe the planned target difference of four points. Our post-hoc sensitivity analysis, accounting for missing data, gave similar results, providing reassurance that our conclusions are robust.

### Conclusion 

The ARTISAN trial has showed that routinely referring patients to a programme of physiotherapy is not superior to a single session of advice, supporting materials, and option to self-refer to physiotherapy. Further research should be directed towards optimising self-management strategies.

**What is already known on this topic**

Non-operative rehabilitation is the most common management following a first time shoulder dislocation
Non-operative management ranges from an advice sheet only, to a programme of individually tailored physiotherapy, typically over a six month period
No previous randomised controlled trial evidence was available to inform the best approach to non-operative rehabilitation

**What this study adds**

ARTISAN is the largest trial on this topic to date, recruiting 482 participants from 40 sites in the UK
An individually tailored programme of physiotherapy compared with a single session of advice, supporting materials, and option to self-refer to physiotherapy did not significantly improve function six months after treatment
Knowing that an individually tailored programme of physiotherapy is not superior will enable clinicians and patients to have evidenced informed discussions about the best approach to non-operative rehabilitation

## Data Availability

All data requests should be submitted to WCTUDataAccess@warwick.ac.uk for consideration. Access to anonymised data may be granted following review.

## Web extra

Extra material supplied by authors

Web appendix: Extra material supplied by authors

## Notes

Contributors: RSK, DE, HP, JM, SD, CM, HB, DT, and MU were responsible for study conception, trial design, obtaining grant funding, and trial management. RSK was responsible for acquisition of data. HP and AH were responsible for the statistical analysis. JM and HN were responsible for the health economics analysis. All authors were responsible for the interpretation of the data and for drafting and approving the final submitted manuscript. RSK had full access to all the data in the study and takes responsibility for the integrity of the data and the accuracy of the data analysis. HP and AH conducted and were responsible for the data analysis. RSK is the guarantor and accepts full responsibility for the work and the conduct of the study and controlled the decision to publish. The corresponding author attests that all listed authors meet authorship criteria and that no others meeting the criteria have been omitted. The corresponding author had full access to all the data in the study and had final responsibility for the decision to submit for publication.

RSK (the manuscript’s guarantor) affirms that the manuscript is an honest, accurate, and transparent account of the study being reported; that no important aspects of the study have been omitted; and that any discrepancies from the study as originally planned have been explained.

Dissemination to participants and related patient and public communities: A lay summary of the results of the study will be made available to research participants on the trial website.

Provenance and peer review: Not commissioned; externally peer reviewed.
