# Supplementary material for: Acute rehabilitation following traumatic anterior shoulder dislocation (ARTISAN): pragmatic, multicentre, randomised controlled trial
Source: BMJ. 2024 Jan 17;384:e076925. doi: 10.1136/bmj-2023-076925 (PMC10792684; doi:10.1136/bmj-2023-076925)
Supplement: Supplementary file 1 — Web appendix: Extra material supplied by authors [file kear076925.ww.pdf]

Supplement two additional sensitivity, subgroup and secondary analysis

Figures

Figure 1: Diagram of participant pathway for the ARTISAN only group

Figure 2: Diagram of physical therapy pathways for the ARTISAN Plus group

Figure 3: Age group cut point

Tables

Table 1: Additional intervention details: Core ARTISAN advice session details

Table 2: Additional physical therapy delivered during additional session

Table 3: Post hoc per protocol (additional physical therapy received)

Table 4: Pre-Specified subgroup: Dominant arm

Table 5: Pre-Specified subgroup: Age group

Figure 1: Diagram of participant pathway for the ARTISAN only group

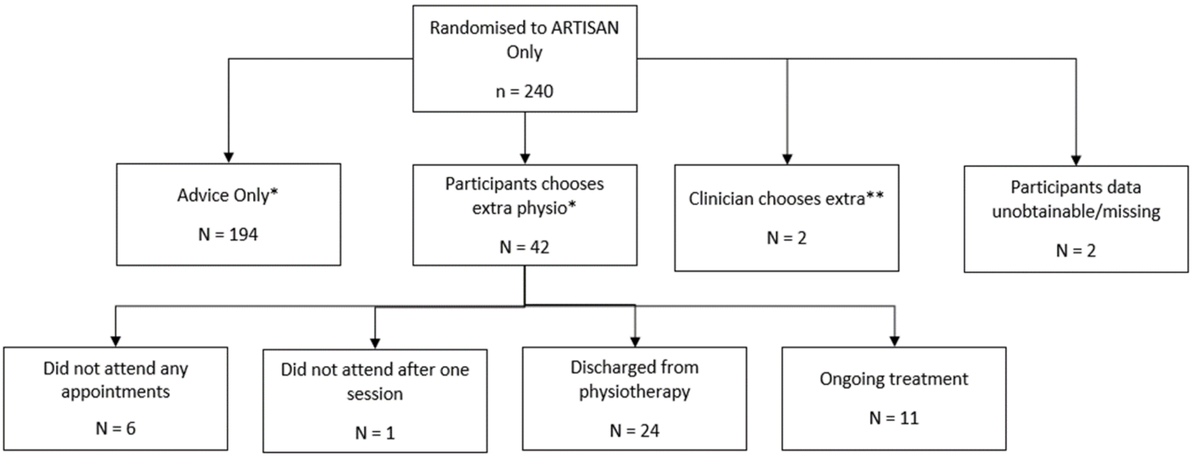

**Participants are compliant with protocol. **Participants are not compliant with protocol (i.e. have switched treatment groups)*

Figure 2: Diagram of physical therapy pathways for the ARTISAN Plus group

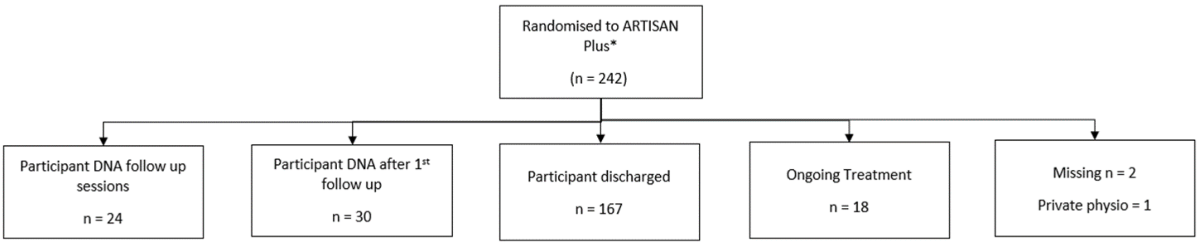

Figure 3: Age group cut point

Due to the uncertainty around the appropriateness of the age cut point, a Gaussian mixture model with a support of two was used to identify the best fit of the ages of the randomized participants. The age where the probability of membership in either distribution was equal was at 33 years of age. As this was within 10 years of the randomisation stratum (age of 40), no further sensitivity analyes exploring the effects of age were conducted. Figure 3 shows probability density plots of the participant age. Two distributions were fit, centred at 24 and 56 years old, with the point where probability of membership in both distributions was 50% at 33 years of age

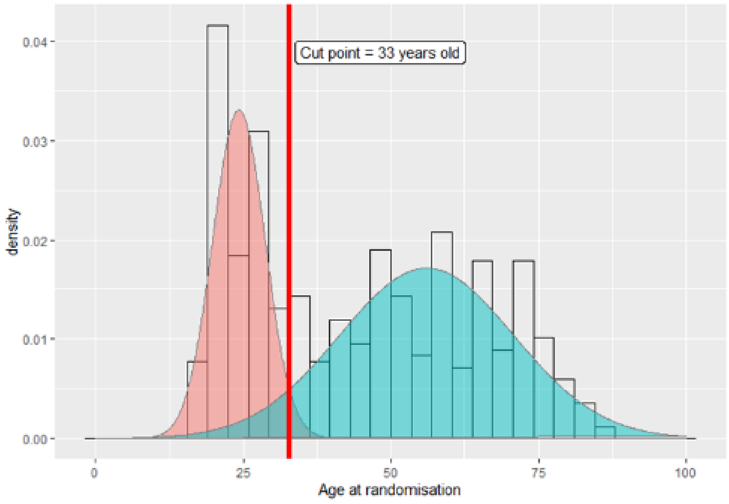

Table 1: Additional intervention details: Core ARTISAN advice session details

Throughout the ARTISAN study, 96 therapists delivered the core ARTISAN session across the 40 study sites. The median number of therapists at each site was 2 and ranged from 1 to 7. The minimum number of participants randomized by each therapist ranged from 1 to 21 randomisations; and of the 96 therapists, only 42 (44%) randomized more than three participants

At the interim analysis point, an estimate of the therapist effect by calculating the intra-cluster correlation coefficient (ICC) using the 3-month follow-up data was estimated using a multi-level model (MLM). A χ^2 test comparing the likelihood of the full model against the model without the physiotherapist effect was also conducted. The ICC was estimated to be 0.0201 with 95% CI 0 – 0.601 (Participants n = 138; physiotherapists N= 67). The addition of physical therapy effects did not improve the model (χ^2 = -2.27e-13, df = 1, P = 1).

A sensitivity analysis with those physiotherapists randomising three or fewer participants were removed. Again, the addition of therapist effects did not improve the model (χ^2 = 1.14e-13, df = 1, P = 0.5). The ICC for was estimated to be 0.0019 with 95% CI 0 – 0.483 (Participants n = 94; physiotherapists N= 23).

The analysis was repeated using the six-month follow up data at the end of the study to check if the interim analysis was correct. Again, 12 sites had a single physiotherapist performing the randomisation of the participants into the ARTISAN study, and many physiotherapists still did not randomise more than three participants.

Repeating the interim analysis using the final 6-month up data showed that the physiotherapist effect was small and statistically insignificant. The model was adjusted for if the dominant arm was injured, age group and physiotherapist, but did not include the allocation group (354 participants and 80 physiotherapists). An ICC value of 0.026 was observed with 95% confidence interval of 0 – 0.106, was observed. Again, comparing the models with and without the physical therapy effects had χ^2 = 0.638 and p-value = 0.424. Again, this showed that including physical therapy effects did not improve the model fit.

Details of the core (pre-randomisation) ARTISAN advice session are given in eTable1.Four hundred and sixty participants were given as per protocol, with 22 participants core ARTISAN session details missing. The mean time taken to deliver the ARTISAN session were similar in both interventions.

Table 1 also contains the self-reported grade of the physiotherapists who delivered the additional physical therapy sessions. (*Note that 22 participant's data were missing).

Table 1: Core ARTISAN advice session details

		**Advice only (n = 240)**	**Advice and physical therapy (n= 242)**	**All participants (n = 482)**
ARTISAN session given as per protocol (therapist report)*		230	230	460
Time taken to deliver ARTISAN session (mins) (mean, SD)		40 (13)	38 (13)	39 (13)
Session longer than 60 minutes		7 (3)	4 (2)	11 (2)
Grade of physiotherapist delivering follow up sessions (at each session)	Grade 5		3	
	Grade 6		115	
	Grade 7		178	
	Grade 8		50	
	Other		4	

Table 2: Additional physical therapy delivered during additional sessions

Exercise	Times given at sessions (n)	Times prescribed for home (n)
Flexion in lying	22	19
Flexion with a stick	12	15
Flexion with a table	23	23
Flexion with a gym ball	4	7
Flexion with a wall	32	36
Flexion with a pulley	5	3
Abduction in lying	4	3
Abduction with a stick	28	26
Abduction with a table	11	11
Abduction with a gym ball	4	3
Abduction with a wall	11	11
Abduction with a pulley	3	3
External rotation in lying	6	5
External rotation with a stick	23	25
Rotation with a table	4	5
Rotation with a gym ball	1	2
Internal rotation with a stick	7	8
Internal rotation with a towel	10	12
Extension with a stick	2	2
Pendula exercises	0	1
Other range of movement exercise	19	26
Thera-band extension	13	16
Thera-band external rotation (standing)	21	24
Thera-band external rotation (sitting)	14	16
Thera-band internal rotation (standing)	20	19
Thera-band internal rotation (sitting)	7	8
Thera-band flexion (stable surface)	14	13
Thera-band flexion (standing)	17	23
Thera-band abduction	24	22
Scapula setting	3	2
Glenohumeral control	22	21
Standing weight drop	16	23
Lying weight drop	3	4
Other strength exercise	99	109
Adv1: floor push ups	22	20
Wall push ups	40	35
Gym ball push ups	3	4
Gym ball weight transfer	5	11
Gym ball proprioception	0	1
Proprioception	1	2
Sport specific drills	5	5
Falling press up, waist level	6	7
Falling press up, standing height	0	2
Other advice	33	49

Table 3: Post hoc per protocol (additional physical therapy received)

In this scenario, we only consider the intervention to be the receipt or not of additional physical therapy sessions. That is, the comparison of those participants in the advice only group who did not have further physical therapy session with participants in the advice and additional physical therapy group who received at least one further session of physical therapy.

Repeating the primary analysis with these participants yielded a model which did not converge. However, including site as a fixed effect showed no significant differences between the two interventions, and broadly similar model coefficients, as shown in eTable3

Model variable (N= 292)		Coefficient	95% CI	p-value
Intervention Group	Advice Only<br>Advice and physical therapy	0<br>0.8	-<br>(-1.2 to 2.7)	0.43
Age group	40 & under<br>Over 40	0<br>-2.4	-<br>(-4.5 to -0.3)	0.02
Dominant Arm injured	Non-Dominant<br>Dominant	0<br>-1.0	-<br>(-3.0 to 0.9)	0.31
Oxford Shoulder Instability	Baseline score	0.4	(0.3 to 0.5)	<0.001

Table 4: Pre-Specified subgroup analysis : Dominant arm

The study pre-specified a subgroup analysis to explore if there is evidence of differences in the intervention effects between participants who injured their dominant arm and non-dominant arm. This was explored by adding an interaction term between the allocation group and dominant arm injury term in the ITT model. The results show that there were no significant differences, nor was the model largely altered.

Model variable (N= 337)		Coefficient	95% CI	p-value
Intervention Group	Advice Only<br>Advice and physical therapy	0<br>0.6	-<br>(2.2 to 3.5)	0.66
Age group	40 & under<br>Over 40	0<br>-2.8	-<br>(-4.8 to -0.8)	0.10
Dominant Arm injured	Non-Dominant<br>Dominant	0<br>-0.1	(-2.9 to 2.6)	0.92
Interaction term	Advice and physical therapy × dominant arm injured	-1.7	(-5.6 to 2.2)	0.41
Oxford Shoulder Instability	Baseline score	0.4	(0.3 to 0.5)	<0.001

Table 5: Pre-Specified subgroup analysis: Age group

The study also pre-specified a subgroup analysis to explore if there is evidence of differences in the intervention effects between the two participant age groups. Again, this was explored by adding an interaction term between the allocation and age groups.

The interaction model for age group showed that older participants who were in the additional physical therapy arm had a small decline in mean function by 0.64 points over those participants allocate to advice only. However, the younger participants mean scores increased by 3.8 points when receiving further physiotherapy compared to advice only. However, this is not a statistically significant or clinically meaningful effect in either case.

Model variable (N= 337)		Coefficient	95% CI	p-value
Intervention Group	Advice Only<br>Advice and physical therapy	0<br>3.8	-<br>(0.6 to 6.9)	0.02
Age group	40 & under<br>Over 40	0<br>-1.0	-<br>(-3.8 to 1.8)	0.48
Dominant Arm injured	Non-Dominant<br>Dominant	0<br>-0.8	-<br>(-2.7 to 1.1)	0.40
Interaction term	Advice and physical therapy and Age group 40 and over	-3.44	(-7.4 to 0.5)	0.09
Oxford Shoulder Instability	Baseline score	0.4	(0.3 to 0.5)	<0.001
